# DAP5 increases axonal outgrowth of hippocampal neurons by enhancing the cap-independent translation of DSCR1.4 mRNA

**DOI:** 10.1038/s41419-018-1299-x

**Published:** 2019-01-18

**Authors:** Ji-Young Seo, Youngseob Jung, Do-Yeon Kim, Hye Guk Ryu, Juhyun Lee, Sung Wook Kim, Kyong-Tai Kim

**Affiliations:** 1 Division of Integrative Biosciences and Biotechnology, Pohang University of Science and Technology (POSTECH), Pohang, Gyeongbuk Republic of Korea; 2 Department of Pharmacology, School of Dentistry, Kyungpook National University (KNU), Daegu, Republic of Korea; 3 Department of Life Sciences, Pohang University of Science and Technology (POSTECH), Pohang, Gyeongbuk Republic of Korea

## Abstract

Proper wiring between neurons is indispensable for proper brain function. From the early developmental stage, axons grow and navigate to connect to targets according to specific guidance cues. The accuracy of axonal outgrowth and navigation are controlled by a variety of genes, and mutations and/or deficiencies in these genes are closely related to several brain disorders, such as autism. *DSCR1* is one of these genes and regulates actin filament formation in axons. Thus, identifying the detailed regulatory mechanisms of DSCR1 expression is crucial for the understanding of the axon development of neurons; however, these regulatory mechanisms of DSCR1 remain unknown. Here, we discovered that mRNA encoding the DSCR1 isoform DSCR1.4 is present and mainly translated by the cap-independent initiation mechanisms in both the soma and axons of hippocampal neurons. We found that translation of *DSCR1.4* mRNA is enhanced by death-associated protein 5 (DAP5), which can bind to *DSCR1.4* 5′UTR. BDNF-stimulus induced an increase in DAP5 expression and the cap-independent translation efficiency of *DSCR1.4* mRNA in axon as well as soma. Furthermore, we showed the importance of the cap-independent translation of *DSCR1.4* on enhancement of DSCR1.4 expression by BDNF-stimulus and axonal outgrowth of hippocampal neurons. Our findings suggest a new translational regulatory mechanism for DSCR1.4 expressions and a novel function of DAP5 as a positive regulator of *DSCR1.4* mRNA translation induced in soma and axon of hippocampal neurons.

## Introduction

Down syndrome candidate region 1 (DSCR1), also known as regulator of calcineurin 1 (RCAN1) regulates calcineurin and has two major isoforms, isoform 1 (DSCR1.1) and isoform 4 (DSCR1.4)^1^. DSCR1.1 and DSCR1.4 are differentially expressed by alternative promoter usage, leading to differences in both the 5′-untranslated region (5′-UTR) of their mRNAs and the N-terminal domain of the polypeptides. DSCR1 localizes in the axons and soma of neurons and controls axonal outgrowth by regulating calcineurin, which dephosphorylates cofilin^2^. Hippocampal neurons in DSCR1-knockout mice have short-axon length. Furthermore, DSCR1 controls local translation in dendritic spines and axon termini^2,3^. Thus, elucidating the regulatory mechanisms of DSCR1 expression in neurons is crucial to understanding normal brain function. Previous studies have described transcriptional and post-translational regulatory mechanisms of DSCR1^4–6^. However, most of these studies utilized non-neuronal cells and did not examine the post-transcriptional regulatory mechanisms of *DSCR1* mRNA.

In eukaryotes, mRNA translation is predominantly initiated by recognition of the m^7^G cap structure at the 5′-UTR^7^. However, it has been reported that translation of some mRNAs involves cap-independent initiation^8^. The mechanism of cap-independent initiation was first elucidated in picornavirus and has also been reported in eukaryotic cells^9,10^. Several genes, such as *p53*, and *Apaf-1*, which are involved in cellular stress responses, require a cap-independent initiation mechanism because cap-dependent translation is blocked under cellular stress^11–13^. However, recent studies demonstrated that even though cap-dependent translation functions well, cap-independent translation is crucial for normal physiologic processes, including circadian rhythm and development^14,15^. In addition, it has been reported that cap-independent translation is necessary for the synthesis of proteins that regulate axonal outgrowth and dendritic spine structure. Because neurons are highly polarized cells with axons and dendrites that have limited translation machinery components, cap-independent translation may be important for various mRNAs in neurons.

In this study, we discovered the importance of *DSCR1.4* translational regulation in neurons and elucidated the regulatory mechanism. Translation of *DSCR1.4* involves both cap-dependent and cap-independent initiation. We identified cis-regulatory elements in the *DSCR1.4* 5′-UTR and a regulator for cap-independent translation known as death-associated protein 5 (DAP5). DAP5 positively regulates *DSCR1.4* mRNA translation. We also confirmed that cap-independent translation of *DSCR1.4* occurs in the axons and soma of neurons. Cap-independent translation of *DSCR1.4* was enhanced in hippocampal neurons treated with brain-derived neurotrophic factor (BDNF). Moreover, our study demonstrated that a reduction in DAP5 leads to a decrease in DSCR1.4 expression and axon length. These findings enhance our understanding of the diverse regulatory mechanisms of finely tuned gene expression in neurons as well as the functions of DAP5 and DSCR1.4.

## Materials and methods

### Animals

All animal experiments were approved by the Pohang University of Science and Technology Institutional Animal Care and Use Committee (POSTECH IACUC) (Approval ID: POSTECH-2015-0051). Used ICR strain mice were purchased from Hyochang Science.

### Cell culture and transient transfection

Mouse neuroblastoma N2A and human neuroblastoma SHSY5Y cells were cultured in Dulbecco’s Modified Eagle’s medium (DMEM; Hyclone) and Minimum Essential Medium (MEM; Hyclone), respectively, supplemented with 10% fetal bovine serum (FBS; Hyclone) and 1% penicillin/streptomycin. Neuroblastoma cells were incubated in 5% CO_2_ at 37 °C. siRNAs and Flag, EGFP tag vectors were transfected into N2A and SHSY5Y cells using the Neon microporation system (Invitrogen). At 24 h after this transfection, transfection of the pRF vector was performed by using Lipofectamine 2000 (Invitrogen) following manufacturer’s instructions. Cells were harvested after 24 h incubation.

Hippocampi were dissected from E17 mouse embryos and treated with DNase and trypsin at 37 °C. Hippocampal primary neurons were seeded on 12-well plate with round glass coverslips or 6-well plate without round glass coverslips coated with poly-l-lysine (Sigma). Neurons were cultured in neurobasal medium with 1% glutamax, 1% penicillin/streptomycin, and B27 supplement. Neurons at DIV 2 or DIV 3 were transfected using Lipofectamine 2000 (Invitrogen) according to manufacturer’s protocol. Neurons were incubated with 30 ng/ml BDNF (PEPROTECH) for 30 min.

### Axon and cell body isolation

For axon and soma isolation, modified Boyden’s chambers were used as previously described^16^. In brief, hippocampal primary neurons were seeded on 6-well plate containing a tissue culture insert with 8 μm polyethylene  terephthalate membrane-coated with poly-l-lysine and laminin. We washed the upper and lower surface of inserts with PBS. The upper surface was scraped several times with cotton applicators to isolate axon compartment at lower surface and the lower surface was scraped in the same way to isolate cell body at upper surface. The insert membrane was removed by a scalpel.

### Plasmids and RNA interference

Bicistronic pRF DSCR1.4 5′UTR, ΔCMV RF DSCR1.4 5′UTR, and hp pRF DSCR1.4 5′UTR vectors for reporter assay were made by inserting the human DSCR1.4 (Accession no. NM_203418.1) or mouse DSCR1.4 (Accession no. NC_000082.6) 5′UTR. mDSCR1.4 5′UTR and hDSCR1.4 5′UTR were PCR-amplified by cDNA derived from N2A and SHSY5Y cells using primers as follows: hDSCR1.4 5′UTR forward primer 5′-AAGTCGACTGTCTGCCTGCAAGCATGC-3′, reverse primer 5′-GGGCTTGCTTTC TTACAGTGAAAG-3′, mDSCR1.4 5′UTR forward primers 5′- AAGTCGACCGTCTGCCCGAGGGCAT GC-3′, reverse primer 5′-GGGTCTGCTTTTTCACGGGGC-3′ (Macrogen, Seoul, Republic of Korea). The amplified PCR products were digested with *Sal1* and *Sma1* enzyme and inserted into intercistronic region of pRF vector. Serially deleted constructs of hDSCR1.4 and mDSCR1.4 5′UTR were made as mentioned above. PCR products amplified for pSK DSCR1.4 5′UTR vector for in vitro binding assay were digested with *EcoR1* and *Xba1* and then inserted into intercistronic region of pSK vector. Bicistronic pRF p53 5′UTR, pRF EMCV 5′UTR, pRF β-globin 5′UTR used as positive and negative control were made in an identical manner by using primers as follows: p53 5′UTR forward primer 5′-AAAAGCTTATGTCGCGGAGGCTGCTGC-3′, reverse primer 5′-CCGGATCCTTAGGAGGC GTGCTGAGC-3′, EMCV 5′UTR forward primer 5′-AAAGTCGACTAACGTTACTGGCCGAAGCC-3′, reverse primer 5′-CCCCCCGGGTGCCATATTATCATCGTGTTTTT-3′, β-globin 5′UTR forward primer 5′-AAGTCGACACATTTGCTTCTGACACA-3′, reverse primer 5′- GGGGGTGTCTGTTTGAGGTTGC-3′

The used siRNA duplex is as follows: Control 5′-CCUACGCCACCAAUUUCGUdTdT-3′ (Bioneer, Daejeon, South Korea), DSCR1.4 #1 5′-GAUGAUGUCUUCAGCGAAdTdT-3′ (Bioneer), DSCR1.4 #2 5′-CUGUGUGGCAAACAGUGAUdTdT-3′, DAP5 #1 5′-AAUGUGGGUGUAGAGUCUAAA-3′ (Bioneer) and DAP5 #2 5′-AAC CAG AGU CAG GGA CUC UUA-3′ (Bioneer).

### Dual-luciferase reporter assay

After transfection of the pRF vector, cells were harvested and lysed in 50 µl Reporter Lysis 5× buffer (Promega). Luciferase activities of the samples were indicated by Dual-Luciferase Reporter Assay System (Promega) and luminometer following the manufacturer’s instructions.

### In vitro binding assay

pSK vectors containing hDSCR Full, Δ54, Δ119, Mut 5′UTR and mDSCR Full, Δ136 5′UTR were linearized with *Xba*I restriction enzyme. Biotin-UTP-conjugated transcripts were transcribed by T7 polymerase and treated with DNase I (Promega). The biotin-conjugated transcripts were incubated with N2A, SHSY5Y cell or hippocampal primary neuron extracts for 30 min at room temperature and then incubated with streptavidin beads (Thermo Scientific) at 4 °C overnight. Proteins interacting with biotin-conjugated transcripts were detected by Western blotting.

### Cell extract preparation and immunoblotting

Harvested cells were lysed with TNE buffer (50 mM Tris, 140 mM NaCl, 5 mM EDTA) containing Pierce^TM^ Protease Inhibitor (Thermo Scientific) by using sonicator. Nuclear/cytosolic fractions of cells for in vitro binding assay were obtained as previously described^11^. Obtained proteins were denatured and separated on 12% sodium dodecyl sulfate polyacrylamide gel electrophoresis gel and then transferred to nitrocellulose membrane. Proteins were detected by monoclonal anti-FLUC(Abcam), anti-DAP5 (Abcam), polyclonal anti-DSCR1 (Sigma-Aldrich), anti-GAPDH (Millipore), anti-Flag (Cell signaling), anti-Lamin B (Santa Cruz), and Horseradish peroxidase (HRP)-conjugated mouse (Thermo Scientific) and rabbit (Promega) secondary antibodies. Enhanced chemiluminescence (ECL) signals were visualized with a LAS-4000 system (FUJI FILM).

### Quantitative real-time RT-PCR

To isolate total RNA from harvested cells, we used TRI Reagent (Molecular Research Center, Cincinnati, OH, USA). Total RNA was reverse-transcribed using ImProm-IITM Reverse Transcription System (Promega) following manufacturer’s instruction. For qRT-PCR, FastStart Universal SYBR Green Master (Rox) (Roche) was used with StepOnePlus Real-Time PCR System (Applied Biosystems, Carlsbad, CA, USA). The used primers are as follows; for the detection of mouse DSCR1.4, forward 5′- AGCTCCCTGATTGCTTGTGT-3′ and reverse 5′- CTGGAAGGTGGTGTCCTTGT-3′; human DSCR1.4, forward 5′-CTCACTAGGGGCTTGACTGC-3′ and reverse 5′-ATTTGGCCCTGGTTTCACTT-3′; mouse β-actin, forward 5′-GGCACCACACCTTCTACAATG-3′ and reverse 5′-GGGGTGTTGAAGGTCTCAAA C-3′; mouse GAPDH, forward 5′-GCCATCAACGACCCCTTCATT-3′ and reverse 5′-GCTCCTGGA AGATGGTGATGG-3′; mouse MAP2, forward 5′-CCACGTACCTGGAGGTGGTAATG-3′ and reverse 5′-GTGATCTACCCGGGCCTTTG-3′

### Fluorescence in situ hybridization

For DSCR1.4 RNA detection, we made digoxigenin (DIG)-labeled RNA probes. We annealed forward primer 5′-TTAGCTCCCTGATTGCTTGT-3′, reverse primer 5′-CCTGGTCTCACTTTCGCTGA-3′, and inserted the product into pGEM-T vector (Promega) containing T7, SP6 promoters following manufacture’s description. The plasmid was linearized with *ApaI* or *SalI* restriction enzyme. DIG-labeled sense probes or antisense probes were transcribed by T7 or SP6 polymerase, respectively, with DIG-RNA labeling Mix and treated with DNase I.

For DSCR1.4 RNA detection, we used Tyramide signal amplification (TSA). All used materials are RNase-free. DIV 3 mouse hippocampal neurons cultured on-chip glasses were washed in 1× PBS and fixed in 4% formaldehyde for 10 min at RT. Hippocampal neurons were permeabilized in 0.1% Trixon X-100-PBS. After 1× PBS washing, cells were rehydrated for 5 min at RT, in 2× SSC, 50% formamide and hybridized for 2 h at 37 °C by DIG-RNA probe denaturated in 80 °C for 5 min. In 0.1× SSC, 50% formamide, 0.1% NP40, 50 °C, cells were washed for 1 h. After being blocked in TN buffer (0.1 M Tris, 0.15 M NaCl, pH 7.5) with FBS 10% for 30 min, cells were incubated in anti-Tau, anti-DAP5 antibody, HRP conjugated anti-DIG antibody (Roche), at 4 °C, overnight. After washed for 30 min in TNT buffer (0.1 M Tris, 0.15 M NaCl, 0.05% Tween 20, pH 7.5), cells were incubated in Cy3 conjugated TSA Plus working solution (PerkinElmer) for 5 min at RT. Cells were incubated with Alexa 488-conjugated mouse secondary antibody and Alexa 647-conjugated rabbit secondary antibody. Nuclei of cells were stained with Hoechst 33342 and chip glasses were mounted with fluorescent mounting medium (Dako) overnight. Fluorescence signals were visualized by fluorescence.

### Puro-PLA and immunocytochemistry

Hippocampal primary neurons on-chip glass were treated with 5 μM puromycin for 30 min at 37 °C, 5% CO_2_ and washed twice in PBS-MC (1× PBS, 1 mM MgCl_2_, 0.1 mM CaCl_2_). For a negative control, 100 μg/ml anisomycin was treated before puromycin treatment. Cells were fixed in 4% paraformaldehyde (PFA; Sigma-Aldrich)-sucrose for 20 min and permeabilized with 0.5% Triton X-100 solution (Sigma-Aldrich) for 15 min. Duolink PLA reagents (Sigma-Aldrich) were used to detect newly synthesized proteins according to the manufacturer’s instructions. The following antibodies were used for puro-PLA; rabbit monoclonal anti-FLUC (Abcam), anti-DAP5 (Abcam), mouse monoclonal anti-puromycin (Merck). Nuclei of cells were stained with Hoechst 33342 and chip glasses were mounted with fluorescent mounting medium overnight. Duolink PLA signals were visualized by fluorescence microscopy (OLYMPUS FV1000).

### Statistical analyses

All quantitative data are shown as mean ± standard error of the mean from independent experiments. *n* Value is shown in the figure legends. Two-way analysis of variance and Student’s *t* test was done using GraphPad Prism 6. *P* < 0.05 was accepted statistically significant. *P* < 0.05, *P* < 0.01, *P* < 0.001, and *P* < 0.0001 are indicated with *, **, ***, and ****, respectively.

## Results

### *DSCR1.4* mRNA translation involves both cap-dependent and cap-independent initiation

**Fig. 1:**
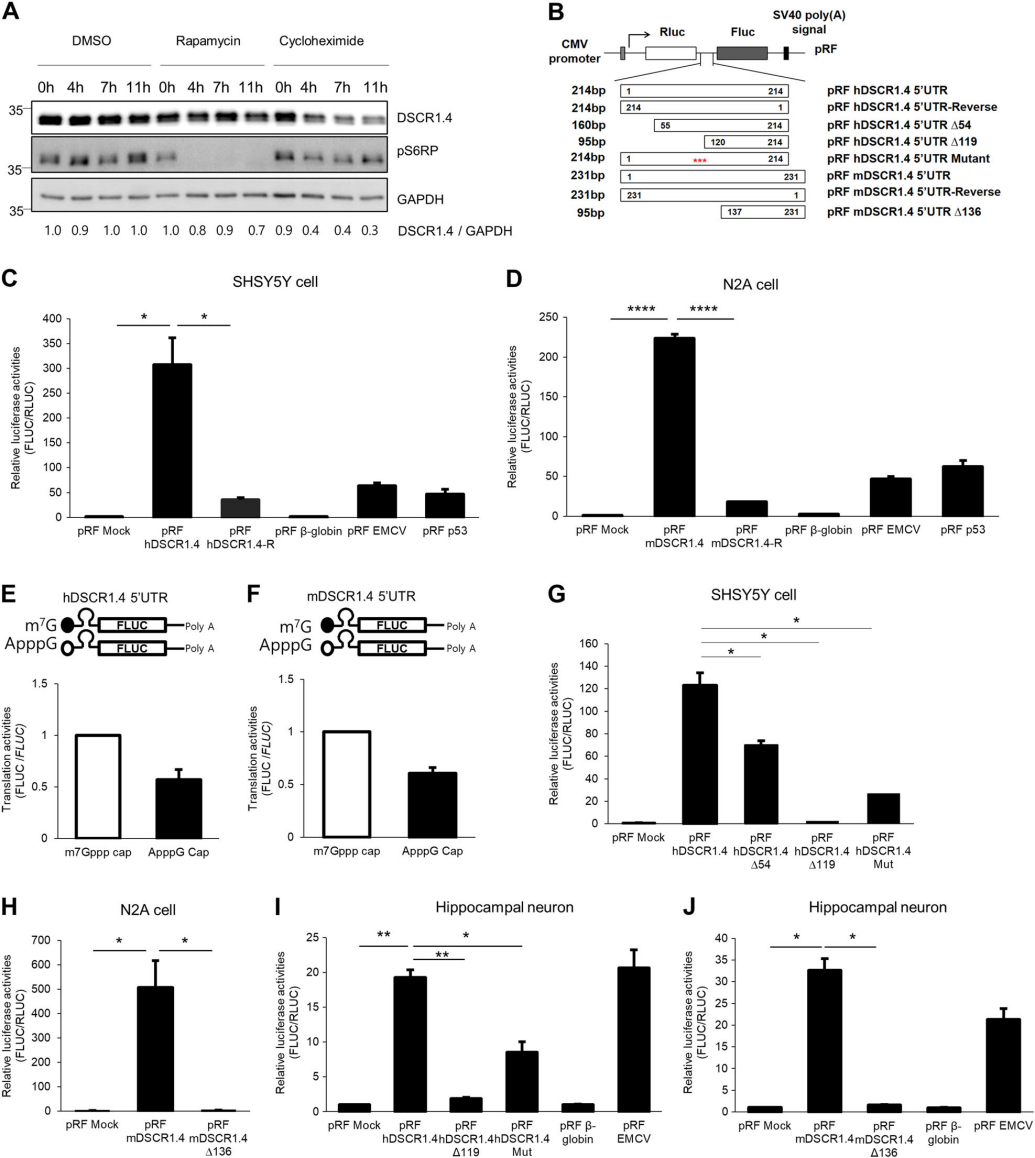
hDSCR1.4 and mDSCR1.4 mRNA are cap-independently translated and have cis-regulatory elements in their 5′UTRs. **a** Cap-independent translational regulation contributes to DSCR1.4 protein expression. SHSY5Y cells were treated with DMSO or 200 μM rapamycin or 50 mg/ml cycloheximide for the indicated times. The levels of endogenous proteins were measured by Western blotting (WB) using anti-DSCR1.4, anti-phosphoS6RP, anti-GAPDH antibodies. GAPDH was used as a loading control. The activity of rapamycin was analyzed by the phosphorylation status of S6RP. The numbers at the bottom indicate the fold increases relative to control. The amount of DSCR1.4 was normalized to GAPDH. **b** Schematic representation of pRF bicistronic luciferase plasmids used for observing cap-independent translation activity of human and mouse DSCR1.4 5′UTR. **c**, **d** hDSCR1.4 5′UTR and mDSCR1.4 5′UTR induce cap-independent translation initiation. **c** SHSY5Y and **d** N2A cells were transfected with the bicistronic reporter plasmids and were incubated for 24 h. pRF β-globin was used as a negative control. pRF EMCV and pRF p53 were used as the positive control. Luciferase activity is shown as the ratio of FLUC to RLUC. Luciferase activity of pRF Mock plasmid transfected cells was set as 1. The bars represent the mean ± SEM (*n* = 3, *n* = 4). **e**, **f** hDSCR1.4 5′UTR and mDSCR1.4 5′UTR prefer cap-independent translation to cap-dependent translation. **e** SHSY5Y cells were transfected with in vitro transcribed m7G capped or ApppG capped hDSCR1.4 5′UTR-FLUC. **f** N2A cells were transfected with in vitro transcribed m7G capped or ApppG capped mDSCR1.4 5′UTR-FLUC. Transfected cells were incubated for 6 h and were harvested. Translation activity is shown as the ratio of FLUC to *FLUC* mRNA. Translation activity of m7G capped transcripts was set as 1. The bars represent the mean ± SEM (*n* = 4, *n* = 3). **g**, **i** The 5′ proximal 119 nucleotides sequence of hDSCR1.4 5′UTR is important for cap-independent translation activity of hDSCR1.4 5′UTR. pRF plasmids with serial deletion constructs and six nucleotides mutant construct were transfected into **g** SHSY5Y cells and **i** mouse primary hippocampal neurons. Luciferase activity is shown as the ratio of FLUC to RLUC. Luciferase activity of pRF Mock plasmid transfected cells was set as 1. The bars represent the mean ± SEM (*n* = 3). **h**, **j** The 5′ proximal 136 nucleotides are essential for cap-independent translation activity of mDSCR1.4 5′UTR. Indicated pRF plasmids were transfected into **h** N2A cells and **j** mouse hippocampal neurons. Luciferase activity of pRF Mock plasmid transfected cells was set as 1. The bars represent the mean ± SEM (*n* = 3, *n* = 3). Data information: In **c**–**j**, ^*^P < 0.05, ^**^P < 0.01, ^****^P < 0.0001 (Student’s *t* test)

Translation initiation is a rate-limiting step in protein production^17,18^. To elucidate the translation initiation mechanism of *DSCR1.4*, we treated SHSY5Y human neuroblastoma cells with dimethyl sulfoxide (DMSO), rapamycin, and cycloheximide. The translation blocker cycloheximide reduced the level of DSCR1.4 protein by 70%, but the cap-dependent translation blocker rapamycin decreased the level of DSCR1.4 protein only by 30% in cells treated separately with these blockers for 11 h (Fig. 1a). We, therefore, hypothesized that cap-independent initiation mechanism is involved in *DSCR1.4* mRNA translation along with cap-dependent initiation mechanism. As almost all cellular mRNAs regulated by cap-independent mechanisms have cis-acting elements in the 5′UTR, we decided to observe the effect of human *DSCR1.4* (*hDSCR1.4*) 5′-UTR and mouse *DSCR1.4* (*mDSCR1.4*) 5′-UTR on the cap-independent translation. First, we analyzed the reported sequences of *hDSCR1.4* 5′UTR and *mDSCR 1.4* 5′UTR to confirm whether they have a similarity (Supplementary Figure 1A–C). As a result, the sequence of *mDSCR1.4* 5′UTR was highly similar to the end sequences of *hDSCR 1.4* 5′UTR. In addition, when we analyzed the genomic sequences of *mDSCR 1.4*, about 136 nucleotides at the upstream of reported 5′UTR of *mDSCR1.4* were highly similar to the nucleotides 1–138 of the *hDSCR1.4* 5′-UTR. Furthermore, because we successfully amplified the region of *mDSCR1.4* from N2A cDNA using oligo dT primers, it is quite possible that the 136 nucleotides at the upstream of 5′UTR of *mDSCR1.4* are also part of the *mDSCR1.4* 5′-UTR, which has yet to be identified. This lead us to question whether the unidentified 136 nucleotides at upstream of *mDSCR 1.4* 5′UTR had effects on the translation of *mDSCR 1.4*, together with reported *mDSCR 1.4* 5′UTR. So for this study, we designated the 231-nucleotide sequence of *mDSCR1.4* containing both the reported *mDSCR1.4* 5′-UTR and 136-nucleotide sequence at upstream of the 5′-UTR as *mDSCR1.4* 5′-UTR and designated 95-nucleotide sequence as *mDSCR1.4* 5′-UTR Δ136. We inserted *hDSCR1.4* 5′UTR, *mDSCR1.4* 5′UTR, and their inverted sequences in between the Renilla luciferase (RLUC) and Firefly luciferase (FLUC) cistrons of a pRF bicistronic reporter vector (Fig. 1b). In pRF bicistronic reporter vector system, RLUC protein is synthesized cap-dependently while FLUC protein is synthesized only when the inserted sequences possess cap-independent translation mechanism. This allows the FLUC/RLUC ratio to represent the cap-independent translation efficiency.

Interestingly, luciferase assay results showed that the *hDSCR1.4* 5′-UTR and *mDSCR1.4* 5′-UTR greatly increased cap-independent translation compared with no insertion (Mock), negative control β-globin 5′UTR, and reversely oriented (Reverse) *hDSCR1.4* 5′-UTR and *mDSCR1.4* 5′-UTR (Fig. 1c, d)^19^. In addition, *hDSCR1.4* 5′UTR and *mDSCR1.4* 5′UTR induced the cap-independent translation activity 4.8-fold and 3.5-fold greater than the positive-control EMCV and p53 5′UTR, respectively^20,21^. These data suggest that *DSCR1.4* mRNA is translated by cap-independent initiation as well as cap-dependent initiation and that these translational mechanisms are conserved in both humans and mice.

Next, to rule out the possibility that FLUC expression was enhanced by a cryptic promoter in the *DSCR1.4* 5′-UTR, *hDSCR1.4* 5′-UTR and *mDSCR1.4* 5′-UTR were inserted into the CMV promoter-deleted pRF (ΔCMV RF) vector (Supplementary Figure 2A). To exclude the possibility of rebinding of ribosomes released from the stop codon of the *RLUC* cistron to the start codon of the *FLUC* cistron, the *hDSCR1.4* 5′-UTR and *mDSCR1.4* 5′-UTR were inserted into a modified pRF vector containing a hairpin structure upstream of *RLUC* (pHRF vector in Supplementary Figure 2B). Promoter deletion and hairpin loop insertion inhibited *RLUC* expression (Supplementary Figure 2C and E). However, the presence of the hairpin loop structure did not block *FLUC* expression, although FLUC activities were dramatically decreased by promoter deletion (Supplementary Figure 2D and F). These results also suggest that *DSCR1.4* mRNA translation in both humans and mice involves cap-independent initiation.

To determine the contribution of cap-independent translation, we constructed transcribed *hDSCR1.4* 5′-UTR-*FLUC* and *mDSCR1.4* 5′-UTR-*FLUC* transcripts having a normal m^7^Gppp Cap or non-functional ApppG cap. Whereas m^7^GpppG-capped mRNAs can be translated in both cap-dependent and cap-independent manner, mRNAs with an ApppG cap can be translated in cap-independent manner. Interestingly, we found that both *hDSCR1.4* 5′-UTR-*FLUC* and *mDSCR1.4* 5′-UTR-*FLUC* mRNAs with an ApppG cap were translated with approximately 60% efficiency compared to mRNAs with an m^7^G cap (Fig. 1e, f). This suggests that cap-independent regulation is preferred for DSCR1.4 protein synthesis.

Next, we tried to identify cis-acting elements in the *DSCR1.4* 5′-UTR responsible for cap-independent translation. As secondary and tertiary structures of mRNA are important for cap-independent translation, we predicted the structure of the *hDSCR1.4* 5′-UTR and *mDSCR1.4* 5′-UTR using the RNA folding program mfold (Supplementary Figure 3A and B)^22^. These results suggested that both *DSCR1.4* 5′-UTRs form three large hairpins. To determine which hairpin structure is critical for the cap-independent translation, we generated pRF vectors with deleted fragments of *hDSCR1.4* 5′-UTR (*hDSCR1.4* 5′-UTR Δ54, Δ119) (Fig. 1b). Luciferase assay results showed that deletions of 54 and 119 nucleotides from the 5′ end decreased cap-independent translation by approximately 40% and 98%, respectively (Fig. 1g). The region between nucleotides 1 and 54 was shown to be important for cap-independent translation of *hDSCR1.4*, but the second loop presented between nucleotide 55 and 119 appeared to be more important.

To investigate the importance of the secondary structure, we disrupted the mRNA structure, particularly the second hairpin loop, without changing the length of 5′-UTR by replacing only 6 nucleotides between nucleotides 95 and 100 (Supplementary Figure 3C). The mutation disrupted the *hDSCR1.4* mRNA structure and significantly suppressed cap-independent translation (Fig. 1g). Moreover, the two 5′-proximal hairpin structures appeared to be important for cap-independent translation of the *mDSCR1.4* 5′-UTR as well, since cap-independent translation activity decreased dramatically following deletion of 136 nucleotides of two proximal hairpin structures (Fig. 1h). The importance of the proximal regions of the *hDSCR1.4* and *mDSCR1.4* 5′-UTRs was also supported by the finding that their 136 5′-proximal nucleotides share approximately 70% sequence identity (Supplementary Figure 1C). These results were also reproduced in mouse hippocampal primary neurons (Fig. 1i, j). Similar result was also observed when in vitro transcribed RF reporter mRNAs were transfected into SHSY5Y or N2A cells to exclude the possibility of production of aberrant mRNA resulting from DNA transfection (Supplementary Figure 3D and E).

### DAP5 protein interacts with the *DSCR1.4* 5′-UTR and enhances cap-independent translation of *DSCR1.4* mRNA

**Fig. 2:**
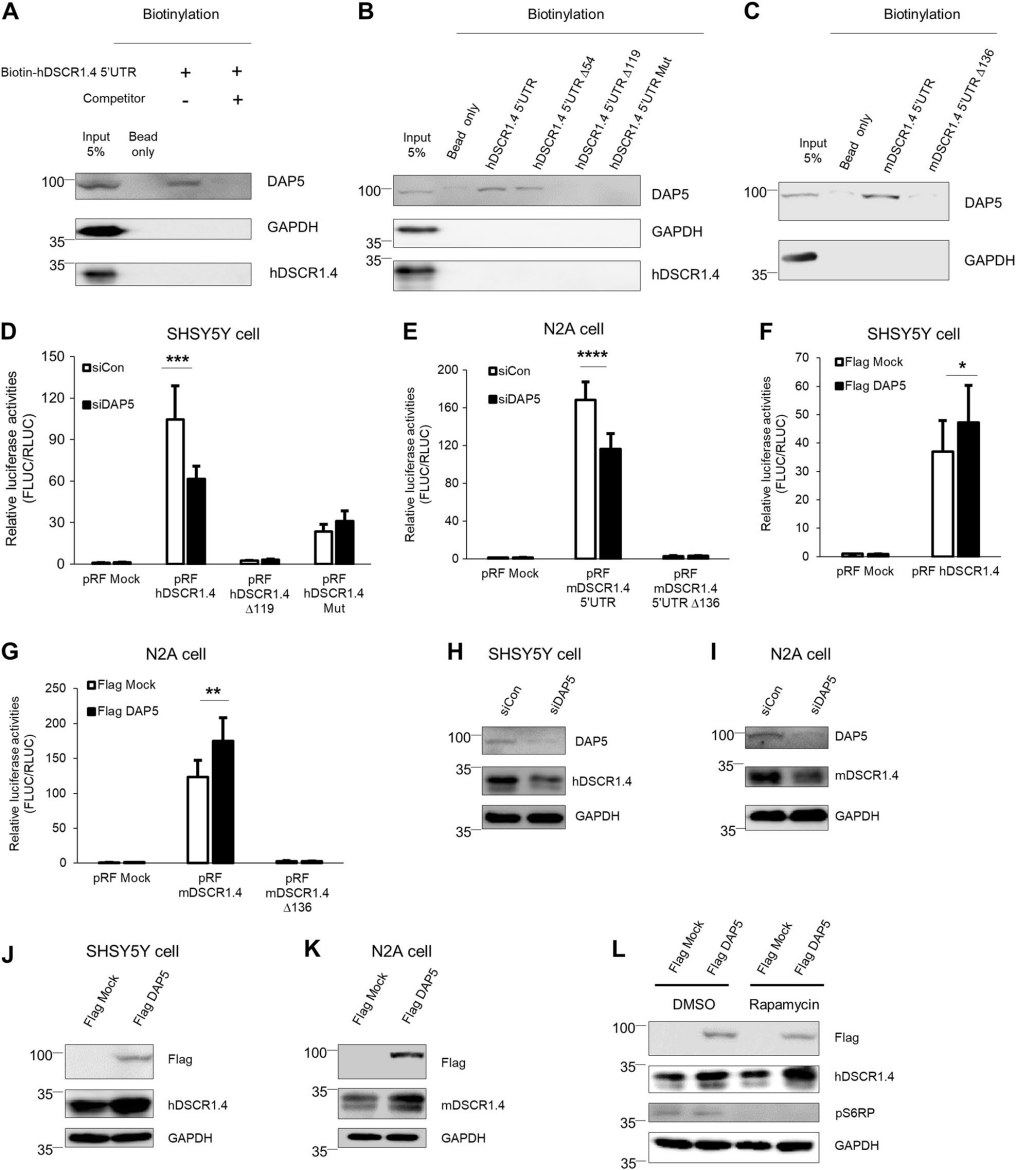
DAP5 positively regulates cap-independent translation of DSCR1.4 by binding to 5′UTR of DSCR1.4. **a**, **b** DAP5 interacts with hDSCR1.4 5′UTR. In vitro transcribed biotin-conjugated hDSCR1.4 5′UTR constructs were incubated with SHSY5Y cell extracts. The region interacting with DAP5 was confirmed by Western blot. GAPDH and hDSCR1.4 were used as negative control. Nonbiotinylated hDSCR1.4 UTR was used as competitor. **c** DAP5 binds to proximal 136 nucleotides of mDSCR1.4 5′UTR. In vitro transcribed biotin-conjugated mDSCR1.4 5′UTR transcripts were incubated with N2A cell extracts. Binding region was verified by Western blot. **d**, **e** A reduction of DAP5 decreases cap-independent translation efficiency of DSCR1.4 5′UTR. **d** SHSY5Y and **e** N2A cells were transfected with control siRNA (siCon) or DAP5 siRNA (siDAP5) and 24 h later with pRF bicistronic vectors. Luciferase activity of pRF mock and siCon transfected cells was set as 1. The bars represent the mean ± SEM (*n* = 7, *n* = 7). **f**, **g** DAP5 overexpression upregulated cap-independent translation of DSCR1.4 5′UTR. **f** SHSY5Y and **g** N2A cells were transfected with Flag Mock or Flag DAP5 plasmids and 24 h later with pRF bicistronic vectors. Luciferase activity of pRF mock and Flag Mock transfected cells was set as 1. The bars represent the mean ± SEM (*n* = 5, *n* = 5). **h**, **i** DAP5 deficiency results in reduction of DSCR1.4 protein levels. siCon or siDAP5 was transfected on **h** SHSY5Y and **i** N2A cells. DAP5 knockdown was confirmed by Western blot using anti-DAP5. **j**, **k** Overexpressed DAP5 increases DSCR1.4 protein levels. **j** SHSY5Y and **k** N2A cells were transfected with Flag Mock or Flag DAP5 and incubated for 24 h. DAP5 overexpression was verified by Western blot using anti-Flag antibody. **l** The increase of DSCR1.4 expressions by DAP5 overexpression results from cap-independent translation. SHSY5Ycells were transfected with Flag Mock or Flag DAP5 and 18 h later incubated and followed by 200 μM rapamycin treatment for 6 h. The rapamycin activity was analyzed by phosphorylation status of S6RP protein. DAP5 overexpression was confirmed by anti-Flag antibody. Data information: In **d**–**g**, *P < 0.05, ^**^P < 0.01, ^***^P < 0.001, ^****^P < 0.0001 (two-way ANOVA)

Cap-independent initiation requires regulatory elements in the 5′-UTR of mRNAs and several protein factors that either induce or inhibit ribosome recruitment. Inhibition and activation of these factors regulates the rate of cap-independent mRNA translation^10^. DAP5 is a well-known regulator that accelerates cap-independent translation of *p53* and *Apaf-1*^23^. Moreover, based on reports that DAP5 is localized in soma and axons, we performed in vitro binding assays to investigate the binding between the *DSCR1.4* 5′-UTR and DAP5 protein^24^. We observed that DAP5 interacted with biotinylated *hDSCR1.4* 5′-UTR and that this interaction was blocked by addition of nonbiotinylated *hDSCR1.4* 5′-UTR, supporting the specificity of this interaction (Fig. 2a). Surprisingly, *hDSCR1.4* 5′-UTRs with 119 proximal nucleotides deletion or 6 nucleotides mutation failed to interact with DAP5. This suggest that the region that interacts with DAP5 lies between nucleotides 55 and 119 of the *hDSCR1.4* 5′-UTR (Fig. 2b). In addition, DAP5 interacted with *mDSCR1.4* 5′-UTR but not with *mDSCR1.4* 5′UTR Δ136 (Fig. 2c). The binding patterns of DAP5 was consistent with cap-independent translation activity of the constructs (Fig. 1i, j), suggesting DAP5 as a positive regulator of cap-independent translation of *DSCR1.4*.

Next, to test the effect of DAP5 on cap-independent translation of *DSCR1.4* mRNA, DAP5 siRNA was transfected into SHSY5Y and N2A cells. DAP5 depletion repressed cap-independent translation of *DSCR1.4* (Fig. 2d, e). Conversely, cap-independent translation of *DSCR1.4* was enhanced by DAP5 overexpression (Fig. 2f, g). However, cap-independent translation mediated by mutated *DSCR1.4* 5′-UTR was not affected by DAP5 depletion. This indicates that the effect of DAP5 on cap-independent translation of the *DSCR1.4* 5′-UTR is very specific and induced by interaction. Consistent with the effect of DAP5 on cap-independent translation of *DSCR1.4*, DAP5 silencing and overexpression evoked a reduction and an increase in endogenous DSCR1.4 protein levels (Fig. 2h–k). However, *DSCR1.4* mRNA was maintained at a constant level regardless of the expression of DAP5 (Supplementary Figure 4A–D). Moreover, even though cap-dependent translation was blocked by rapamycin, DSCR1.4 protein levels increased following the upregulation of DAP5, supporting the hypothesis that DAP5 regulates DSCR1.4 protein synthesis through cap-independent translation (Fig. 2l).

### *DSCR1.4* mRNAs are localized in both the soma and axons and locally translated via a cap-independent mechanism

**Fig. 3:**
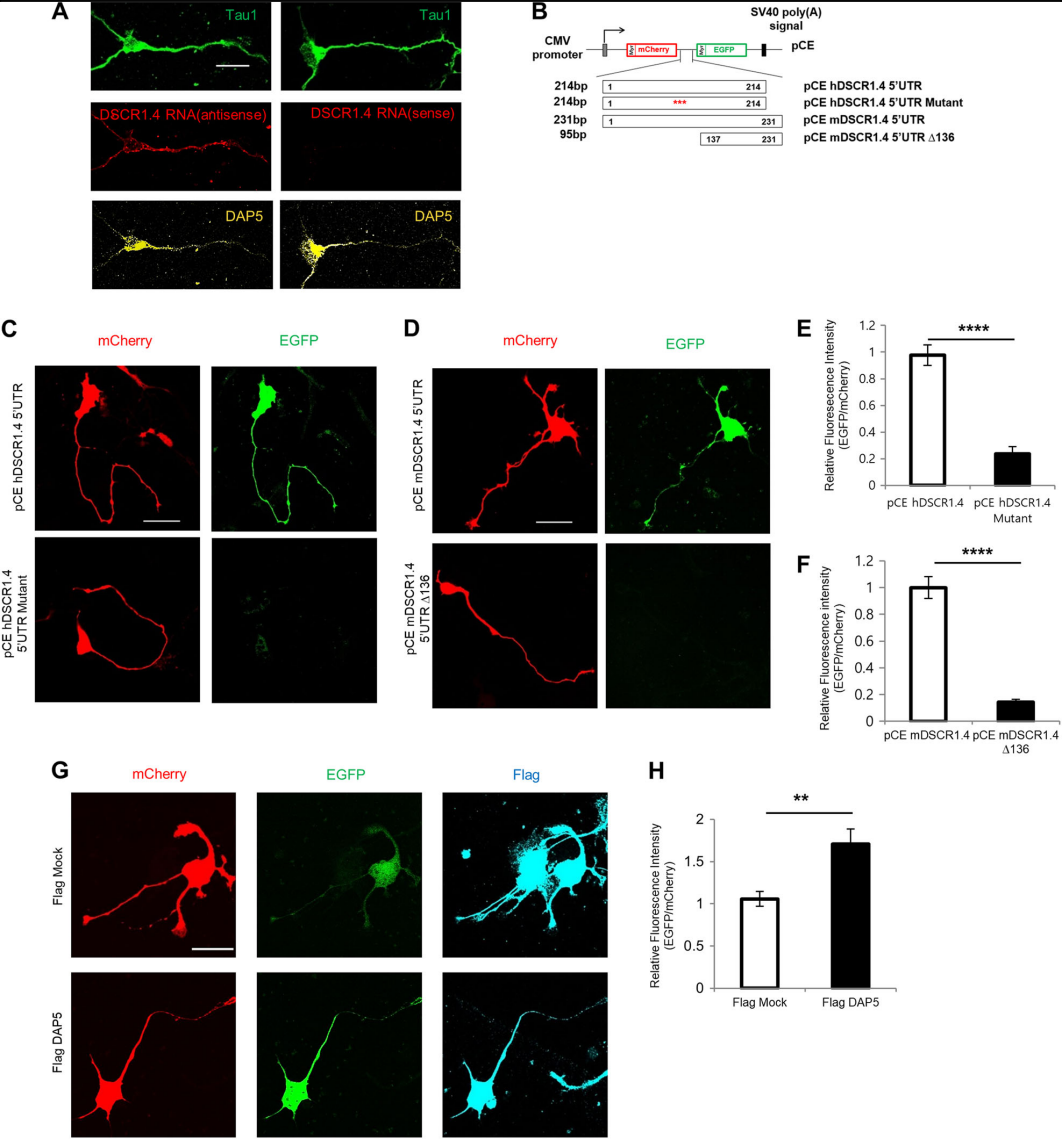
DSCR1.4 mRNAs are localized in both soma and axon and are locally translated in cap-independent manner. **a** DSCR1.4 mRNA and DAP5 protein are localized in the axon as well as the soma. To verify localization of DSCR1.4 mRNA in neurons, TSA-FISH was performed in DIV 3 mouse hippocampal neurons. Tau1 was detected to identify axon. Scale bar, 20 μm. **b** Schematic representation of pCE bicistronic fluorescent plasmids used for observing cap-independent local translation of human and mouse DSCR1.4 mRNA. DSCR1.4 5′UTR constructs were inserted between mCherry and EGFP cistron. DSCR1.4 3′UTR was inserted on upstream of EGFP cistron. **c**–**f** Cap-independent translation of DSCR1.4 mRNA occurs in both axon and soma. pCE bicistronic plasmids were transfected into DIV 2 hippocampal neurons and 24 h later, cells were fixed. **c**, **d** Representative image obtained from confocal microscopy. Scale bar, 30 μm. **e**, **f** The graph shows relative fluorescence intensity measured by Image J. The bars represent the mean ± SEM (pCE hDSCR1.4 5′UTR; *n* = 14, pCE hDSCR1.4 mutant; *n* = 18, pCE mDSCR1.4 5′UTR; *n* = 25, pCE mDSCR1.4 Δ136; *n* = 28). **g**, **h** DAP5 overexpression increases cap-independent translation of DSCR1.4 in both axon and soma. Flag DAP5 and pCE mDSCR1.4 5′UTR plasmids were co-transfected into DIV 2 hippocampal neurons and cells were subjected to immunocytochemistry for Flag in 24 h and DAP5 overexpression was confirmed by Flag antibody. **g** Representative image showing Flag, mCherry, EGFP signal obtained by confocal microscopy. Scale bar, 30 μm. **h** The graph shows relative fluorescence intensity measured by Image J. The bars represent the mean ± SEM (Flag Mock; *n* = 18, Flag DAP5; *n* = 22). Data information: In **e**, **f**, **h**, ^*^P < 0.05, ^****^P < 0.0001 (Student’s *t* test)

To determine where the translation of *DSCR1.4* takes places in neurons, we detected the localization of *DSCR1.4* mRNA using tyramide signal amplification**–**fluorescence in situ hybridization (FISH). The results showed that *DSCR1.4* mRNAs are present in the soma and axons of hippocampal neurons (Fig. 3a). In line with our previous data, DAP5 protein also localized in the soma and axons of hippocampal neurons. This result suggested that cap-independent translation of *DSCR1.4* occurs in axons as well as the soma. To determine regions where cap-independent translation of *DSCR1.4* occurs, we constructed pCE bicistronic vectors in which *DSCR1.4* 5′-UTR constructs were inserted between mCherry and EGFP cistrons with N-terminal myristoylation signals, and the *DSCR1.4* 3′-UTR was inserted at downstream of the EGFP cistron (Fig. 3b)^25^. Because myristoylated mCherry and EGFP rarely diffuse from sites of protein synthesis, their signals indicate sites of protein synthesis. mCherry and EGFP signals indicate cap-dependent and cap-independent translation, respectively. Whereas only mCherry signals were visible in hippocampal neurons with pCE *hDSCR1.4* 5′-UTR mutant or pCE *mDSCR1.4* 5′-UTR Δ136, both mCherry and EGFP signals were detected over the whole area of neurons harboring the pCE *hDSCR1.4* 5′-UTR or pCE *mDSCR1.4* 5′-UTR vector (Fig. 3c–f). These results were consistent with data from previous luciferase assays (Fig. 1g, h) and indicate that cap-independent translation of *DSCR1.4* occurs in neuronal axons and cell bodies. Moreover, DAP5 overexpression enhanced cap-independent translation of *DSCR1.4* in both the soma and axons (Fig. 3g, h).

### Increased DAP5 expression mediated by BDNF accelerates *DSCR1.4* mRNA translation

**Fig. 4:**
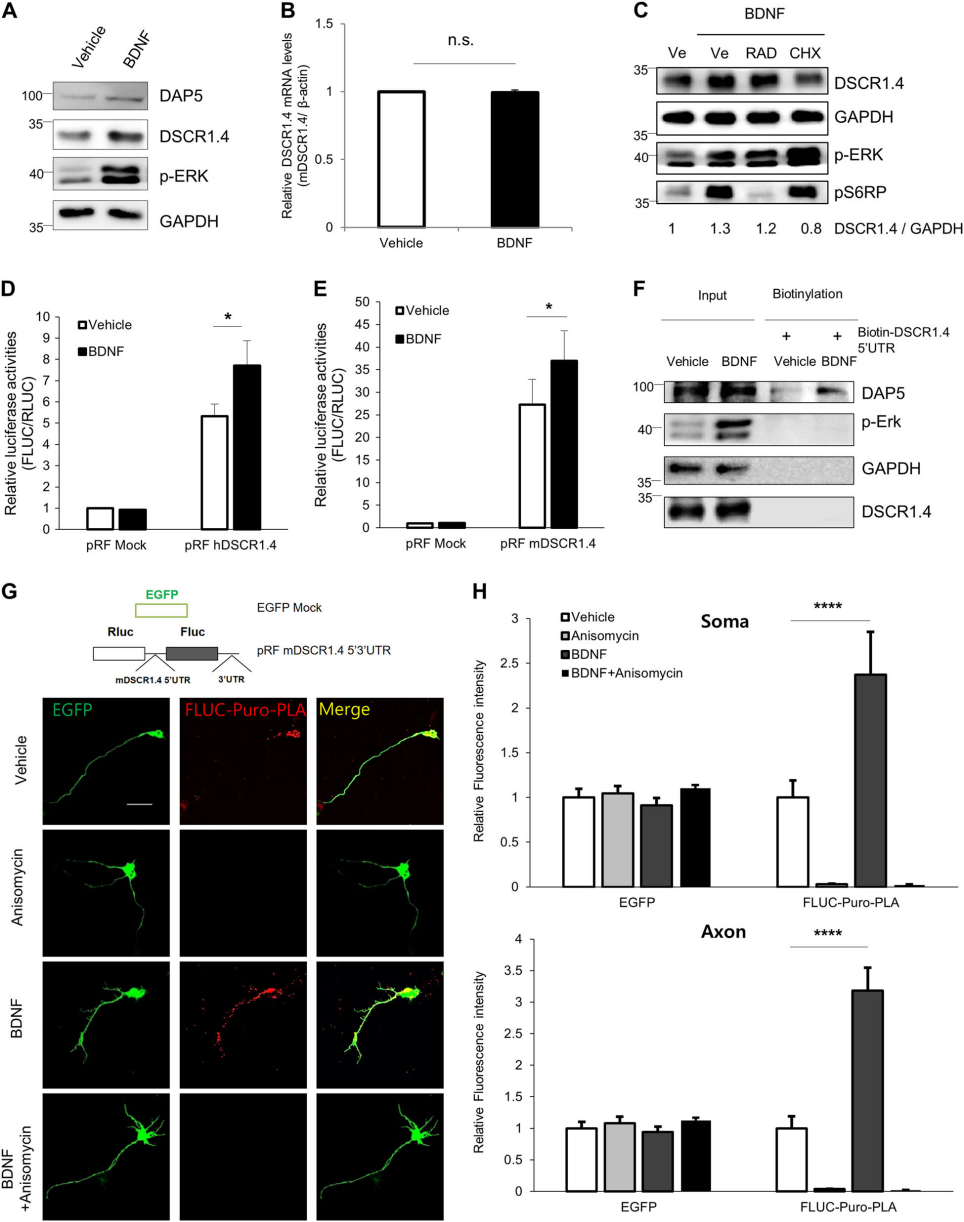
BDNF makes cap-independent translation of DSCR1.4 mRNA more actively by increasing DAP5 expression. **a**, **b** BDNF treatment on DIV 3 hippocampal neuron increases protein levels of DAP5 and DSCR1.4 but not DSCR1.4 mRNA level. Vehicle (DDW) or 30 μM BDNF were treated for 1 h. **a** The protein levels were confirmed by Western blot. GAPDH and phosphorylation of ERK were used as a loading control and marker of BDNF activity, respectively. **b** Endogenous DSCR1.4 mRNA levels were analyzed by qRT-PCR and were normalized to β-actin. The bars represent the mean ± SEM (*n* = 3). **c** Cap-independent translation is essential for DSCR1.4 protein accumulation by BDNF. DIV 3 hippocampal neurons were treated with vehicle (DMSO), 100 μM RAD001(RAD) or 50 mg/ml cycloheximide (CHX) for 3 h followed by BDNF treatment for 1 h. The levels of each protein were confirmed by Western blot. The numbers at the bottom indicate the fold relative to a vehicle. The amount of DSCR1.4 was normalized to GAPDH. **d**, **e** BDNF raises cap-independent translation activity of DSCR1.4 mRNA. At 24 h after **d** pRF hDSCR1.4 5′UTR or **e** pRF mDSCR1.4 5′UTR vectors were transfected into DIV 2 hippocampal neurons, Vehicle (DDW) and BDNF were treated to the neurons for 1 h. The bars represent the mean ± SEM (**e**; *n* = 5, F; *n* = 3). **f** BDNF increases the interaction between DAP5 and DSCR1.4 5′UTR. In vitro transcribed biotin-DSCR1.4 5′UTR was incubated with extracts of the vehicle (DDW) or 30 μM BDNF-treated DIV 3 mouse hippocampal neurons. DAP5 binding was measured by Western blot. Phospho-ERK was used to confirm the activity of BDNF. GADPH was used as a loading control and negative control. **g**, **h** BDNF increases the cap-independent local translation of DSCR1.4 mRNA in axon as well as soma. EGFP and pRF mDSCR1.4 5′ 3′ UTR vectors were co-transfected into DIV 2 mouse hippocampal neurons. At 24 h later, 100 μM anisomycin was treated for 3 h and then 30 μM BDNF was treated for 1 h, followed by 5 μM puromycin treatment for 40 min. To detect newly synthesized FLUC proteins, Puro-PLA assay was conducted. **g** Representative image obtained from confocal microscopy. **h** The graph shows relative fluorescence intensity measured by Image J. The bars represent the mean ± SEM (Vehicle; *n* = 11, Anisomycin; *n* = 12, BDNF; *n* = 11, BDNF + Anisomycin; *n* = 11). Scale bar, 30 μm. Data information: In **d**, **e**, **h**, ^*^P < 0.05, ^****^P < 0.0001 (two-way ANOVA, Student’s *t* test)

Given that *DSCR1.4* mRNA and DAP5 protein are localized in the axons of neurons, we hypothesized that exogenous signals that increase axonal protein synthesis would enhance local translation of *DSCR1.4* in axons. We observed upregulated expression of DSCR1.4 protein (but not of DSCR1.1) in both the soma and the axon of BDNF-treated hippocampal neurons (Fig. 4a and Supplementary Figure 5A, B). The BDNF-induced increase in DSCR1.4 protein was not due to enhanced transcription and RNA stability. Also, BDNF did not change the distribution of *DSCR1.4* mRNA in neurons (Fig. 4b and Supplementary Figure 5C, D). In addition, as the *DSCR1.1* 5′-UTR did not induce cap-independent translation, we hypothesized that the difference between DSCR1.4 and DSCR1.1 protein accumulation under BDNF treatment was the result of cap-independent translation; therefore, cap-independent translation is important for induction of DSCR1.4 (Supplementary Figure 5E). To test this hypothesis, primary hippocampal neurons were treated with cycloheximide or RAD001 and rapamycin, cap-dependent translation blockers, followed by BDNF treatment (Fig. 4c and Supplementary Figure 5F). Cycloheximide inhibited the BDNF-induced increase in DSCR1.4 protein levels, indicating that translation is crucial in the enhancement of DSCR1.4 expression by BDNF. Rapamycin and RAD001 did not block the enhancement of DSCR1.4 expression by BDNF. These data suggest that BDNF mainly enhances DSCR1.4 protein expression by increasing cap-independent translation of *DSCR1.4*. Indeed, we found that BDNF upregulated cap-independent translation of *DSCR1.4* (Fig. 4d, e), perhaps because BDNF enhances both DAP5 expression and the interaction between DAP5 and the *DSCR1.4* 5′-UTR (Fig. 4a, f).

Regions at which de novo cap-independent translation of *DSCR1.4* is induced by BDNF were visualized using a puromycin-proximity ligation assay (Puro-PLA) followed by immunostaining^26^. The pRF *mDSCR1.4* 5′3′-UTR vector was constructed by inserting the *mDSCR1.4* 5′-UTR between the *RLUC* and *FLUC* cistrons and its 3′-UTR at downstream of the *FLUC* cistron of the pRF vector. Detection of *FLUC* puro-PLA puncta indicates cap-independently synthesized protein. pRF *mDSCR1.4* 5′3′-UTR and EGFP vectors were co-transfected into neurons, which were then treated with DMSO or anisomycin, followed by puromycin and BDNF. As expected, *FLUC* puro-PLA puncta were detected in the cell body and axons of puromycin-treated neurons, but no puncta were observed in neurons treated with the translation blocker anisomycin in addition to puromycin. This indicated that *mDSCR1.4* mRNA is translated cap-independently in the soma and axons (Fig. 4g). The staining intensity of *FLUC* puro-PLA puncta increased about 2.4-fold and 3.2-fold in the soma and axon, respectively, of neurons treated with BDNF and puromycin compared with neurons treated with vehicle and puromycin (Fig. 4h). On the other hand, *FLUC* puro-PLA puncta were barely detected regardless of BDNF treatment in neurons transfected with pRF β-globin 5′UTR (Supplementary Figure 5G and H). Based on this result, we wondered whether local synthesis of DAP5 protein is also induced by BDNF stimulation. BDNF was found to increase the translation of DAP5 (Supplementary Figure 5I and J). Thus, we determined that BDNF accelerates the synthesis of DSCR1.4 protein in axons and the soma of neurons by increasing DAP5 expression and enhancing the interaction between DAP5 and *DSCR1.4* mRNA.

### DAP5 is required for axon development in hippocampal neurons

**Fig. 5:**
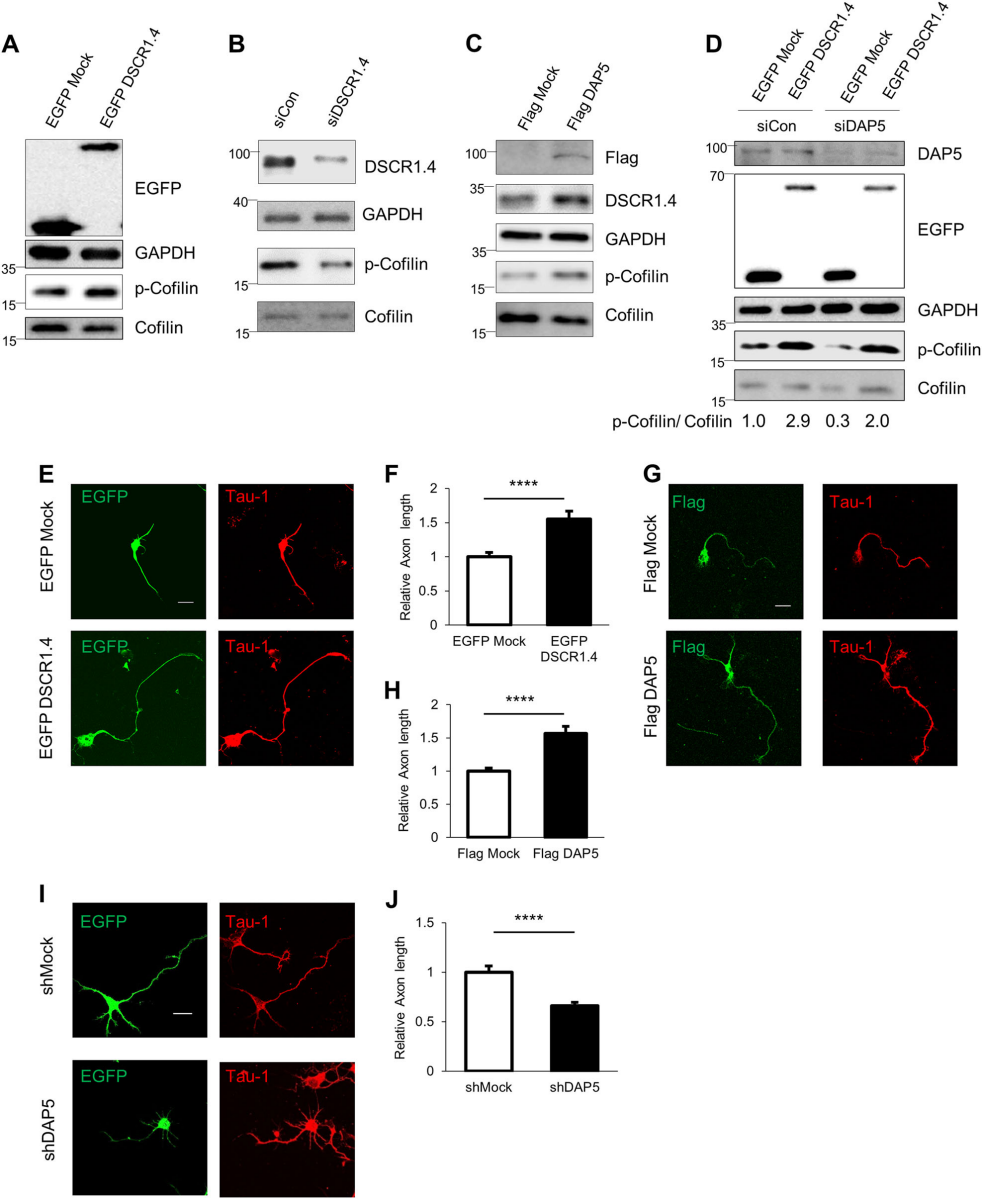
DAP5 regulates axonal outgrowth of hippocampal neurons by enhancing DSCR1.4 expressions. **a** DSCR1.4 overexpression results in the increment of phospho-cofilin/cofilin ratio. EGFP Mock or EGFP DSCR1.4 plasmids were transfected into N2A cells. Phospho-cofilin and cofilin levels were detected by Western blot. DSCR1.4 overexpression was confirmed by anti-EGFP antibody. **b** Inhibition of DSCR1.4 expression reduces phospho-cofilin/cofilin ratio. siCon or siDSCR1.4 were transfected into N2A cells. Phospho-cofilin and cofilin levels were detected by Western blot. Anti-DSCR1 antibody was used to confirm DSCR1.4 knockdown.**c** Increased DAP5 expression raises phospho-cofilin/cofilin ratio by accelerating DSCR1.4 mRNA translation. N2A cells were transfected with Flag Mock or Flag DAP5 vectors and incubated for 24 h. DAP5 overexpression was confirmed by Western blot using anti-Flag antibody. **d** Phospho-cofilin/cofilin ratio was reduced by suppressed DAP5 expression and the reduction was recovered by DSCR1.4 overexpression. N2A cells were co-transfected with designated siRNA and plasmids and incubated for 24 h. The numbers at the bottom indicate the fold relative to siCon and EGFP Mock co-transfected cells. **e**, **f** Increased DSCR1.4 expression facilitates axon outgrowth. EGFP Mock or EGFP DSCR1.4 plasmids were transfected into DIV1 mouse hippocampal neurons. Tau1 was detected to identify axon. **e** Representative image obtained by confocal microscopy. **f** The graph shows relative axon length measured by Image J. The bars represent the mean ± SEM (EGFP Mock; *n* = 25, EGFP DSCR1.4; *n* = 18). **g**, **h** Induction of DAP5 increases axon length. Flag Mock or Flag DAP5 plasmids were transfected into DIV1 mouse hippocampal neurons. **g** Representative image obtained by confocal microscopy. **h** The graph shows relative axon length measured by Image J. The bars represent the mean ± SEM (Flag Mock; *n* = 29, Flag DSCR1.4; *n* = 25). **i** Reduced DAP5 expressions inhibit axon outgrowth. shRNAs were transfected into DIV1 hippocampal neurons. EGFP signal was only detected on cells with shRNAs. **i** Representative image. **j** The graph shows relative axon length measured by Image J. Axon length of shMock plasmid transfected cells was set as 1. The bars represent the mean ± SEM (shMock; *n* = 28, shDAP5; *n* = 26). Scale bar, 20 μm. Data information: In **f**, **h**, **j**, ^****^P < 0.0001 (Student’s *t* test)

The importance of DSCR1 for axon outgrowth was demonstrated by experiments showing that DSCR1-deficient hippocampal neurons have shorter axons. DSCR1 inhibits calcineurin, which dephosphorylates phospho-cofilin to cofilin. Thus, downregulation of DSCR1 expression decreases phospho-cofilin/cofilin ratio, actin polymerization, and axon growth^2^. We confirmed that DSCR1.4 overexpression and DSCR1.4 knockdown result in an increase and decrease in the phospho-cofilin/cofilin ratio, respectively (Fig. 5a, b). Concordantly, an elevated phospho-cofilin/cofilin ratio was observed in cells transfected with Flag-tagged DAP5 (Fig. 5c). A reduction in DAP5 expression lowered the phospho-cofilin/cofilin ratio, presumably by reducing the level of DSCR1.4. The effect of DAP5 knockdown was substantially rescued by transfection with *DSCR1.4* expression plasmid (Fig. 5d), supporting the hypothesis that a reduction in the phospho-cofilin/cofilin ratio due to DAP5 deficiency is a result of a decrease in DSCR1.4 expression.

We measured axon length in DIV 3 hippocampal neurons overexpressing DSCR1.4 or DAP5 (Fig. 5e–h). As expected, when overexpressed, both DAP5 and DSCR1.4 increased axon length. These results are consistent with the previous increase in the phospho-cofilin/cofilin ratio when DSCR1.4 or DAP5 was overexpressed. Conversely, DAP5 knockdown resulted in a reduction in axon length (Fig. 5i, j, and Supplementary Figure 6A). Collectively, these data demonstrate that DAP5 contributes to axonal outgrowth through regulation of the translation of *DSCR1.4*. Furthermore, the observed effect of DAP5 on axon length support the necessity of cap-independent local translation of *DSCR1.4* for axonal outgrowth.

## Discussion

It has been reported that cap-independent translation of cellular mRNAs is important and is enhanced under cellular stress conditions to consistently synthesize proteins even when cap-dependent translation is blocked by cellular stresses. For example, the cap-independent translation efficiency of *p53* and XIAP mRNA increases under DNA-damage and serum deprivation condition, respectively^20,21^. We wondered whether the cap-independent translation activity of *DSCR1.4* mRNA also increases under cellular stress conditions. As a result, the cap-independent translation efficiency did not increase in conditions of etoposide treatment and serum starvation (Supplementary Figure 7A–D). We think that the cap-independent translation of *DSCR1.4* primarily contributes to normal and BDNF stimulated condition but not cellular stress conditions. However, we cannot exclude a possibility that other cell stress conditions might increase the cap-independent translation activity of *DSCR1.4*.

BDNF promoted the expression of DSCR1.4 but not DSCR1.1 (Supplementary Figure 5A). Transcription and mRNA stability were not the contributing factors for this difference, as BDNF treatment did not affect the levels and distribution of *DSCR1.4* mRNA (Fig. 4b). Interestingly, *DSCR1.1* mRNA did not utilize cap-independent initiation (Supplementary Figure 5E). It seems that cap-independent translation activity accounts for this difference in gene expression, as some reports have suggested cap-independent translation as a crucial regulatory mechanism for local translation of various mRNAs induced by synaptic activity in the dendritic spine and guidance cues in axons^27,28^. Because components of the translation machinery are limited in axons, cap-independent translation would be an effective and essential mechanism in axons. Our findings provide new insights into the importance of cap-independent translation, but more research regarding its contribution to other neurophysiologic processes is needed.

DAP5 levels were elevated in cultured hippocampal neurons treated with BDNF (Fig. 4a), and DAP5 translation was dramatically enhanced by BDNF (Supplementary Figure 5I and J). Another study showed that *DAP5* mRNAs exist in axons and the soma and are locally translated, supporting the possibility that BDNF accelerates local synthesis of DAP5 protein^24^. We believe that the cap-independent translation of *DAP5* is enhanced by BDNF stimulation, similar to *DSCR1.4* mRNA, as *DAP5* mRNA can also be translated in a cap-independent manner^29^. In addition, both translational and post-translational regulation of DAP5 in BDNF-treated hippocampal neurons may have an impact on increases in DAP5 protein levels, although we have not investigated this possibility. Further research in this regard will be needed to provide a more detailed understanding of the regulatory mechanism and function of DAP5.

Although DAP5 is known to accelerate the cap-independent translation of various mRNAs, no studies examining the effect of DAP5 on cap-independent translation of mRNAs in neurons have been published. Furthermore, it was reported that DAP5 affects axon length in sympathetic neurons, but the underlying mechanism remained unclear. Here, we found that DAP5 controls cap-independent translation of *DSCR1.4* in the soma and axons, resulting in axonal outgrowth in hippocampal neurons. We expect that our findings will provide a new point of view about the role of DAP5 in diverse physiological processes.

Proper axon guidance is necessary for precise connectivity, and “miswiring” underlies several neurodevelopmental disorders, such as intellectual deficits^30^. *DSCR1* is considered a critical gene for proper wiring through its regulation of neurite outgrowth and growth cone turning. Defects in this gene are closely related to intellectual disorders such as Down syndrome and fragile X syndrome^2,31^. Abnormal expression of DSCR1.4 induces irregular axon outgrowth. Thus, spatio-temporal expression of DSCR1.4 must be finely tuned, but many details of the regulatory mechanism remain to be elucidated. In this respect, our findings of cap-independent translation and elucidation of the regulatory mechanism underlying DSCR1.4 expression have significant meaning. Moreover, the discovery of cap-independent local translation of *DSCR1.4* suggests that DSCR1.4 contributes to various cellular and molecular processes in axon termini of neurons. Therefore, the roles of other trans-acting factors and various physiological conditions in regulating cap-independent translation of *DSCR1.4* deserve further investigation.

## Supplementary information

Supplementary Figure
