# Supplementary material for: DAP5 increases axonal outgrowth of hippocampal neurons by enhancing the cap-independent translation of DSCR1.4 mRNA
Source: Cell Death Dis. 2019 Jan 18;10(2):49. doi: 10.1038/s41419-018-1299-x (PMC6362140; doi:10.1038/s41419-018-1299-x)
Supplement: Supplementary file 1 — Supplementary Figure [file 41419_2018_1299_MOESM1_ESM.pdf]

Supplementary Figure 1. Seo *et al.*

A hDSCR1.4 5'UTR -214nt

1 tgtctgcctg caagcatgca ggacttgact caggaatttg ctgtccaaac aggatgctgt ggaagctgca cttttttttt ccccagggag

91 tggggggctgg cccttactgc tttataagca ccagctcaag aaggaaccta cagcctcttg gaaaggaatc tcactagggg cttgactgcg

181 tgggtctgta gcgctttcac tgtaagaaag caag

B mDSCR1.4 5'UTR -231nt

1 cgtctgcccg agggcatgcg ggacctgact caggaatttg ctgtctaaac aggacgctct ggaagccaag ccctccccgc ccccaggggg

91 ctggctctcc gcggagcttt ttaagctgcg gctggagaaa ggacccgtgg tctcgctcgt tctcttgcaa aggaacctcc agcttgggct

181 tgactgagag agcgagtcgt tcgttaagcg tctgccccgt gaaaaagcag a

C

```
          1       10        20        30        40        50        60        70        80        90        100
          |--------+---------+---------+---------+---------+---------+---------+---------+---------+---------|
Human     TGTCTGCCTGCAAGCATGCAGGACTTGACTCAGGAATTTGCTGTCCAAACAGGATGCTGTGGAAGCTGCACTTTTTTTTTCCCCAGGGAGTGGGGGCTGG
Mouse     CGTCTGCCCGAGGGCATGCGGGACCTGACTCAGGAATTTGCTGTCTAAACAGGACGCTCTGGAAGCCAAGCCCTCCCCGCCCCCAGGGGGCTGGCTCT--
Consensus cGTCTGCCcGaaaGCATGCaGGACcTGACTCAGGAATTTGCTGTCcAAACAGGAcGCTcTGGAAGCcaaaCccTccccgcCCCCAGGGaGcgGGcgCT..

          101     110       120       130       140       150       160       170       180       190       200
          |--------+---------+---------+---------+---------+---------+---------+---------+---------+---------|
Human     CCCTTACTGCTTTATAAGCACCAGCTCAAGAAGGAACC-----TACAGC-----CTCTTGGAAAGGAATCTC-ACTAGGGGCTTGACTGCGTGGGTCTGT
Mouse     -CCGCGGAGCTTTTTAAGCTGCGGCTGGAGAAAGGACCCGTGGTCTCGCTCGTTCTCTTGCAAAGGAACCTCCAGCTTGGGCTTGACTGAGAGAGCGAGT
Consensus .CCgcacaGCTTTaTAAGCacCaGCTcaAGAAaGaACC.....TacaGC.....CTCTTGcAAAGGAAcCTC.AccagGGGCTTGACTGaGaGaGccaGT

          201     210       220       230 234
          |--------+---------+---------+---|
Human     ---------AGCGCTTTCACTGTAAGAAAGCAAG
Mouse     CGTTCGTTAAGCGTCTGCCCCGTGAAAAAGCAGA
Consensus .........AGCGccTgCaCcGTaAaAAAGCAaa
```

Supplementary Figure 1. Human DSCR1.4 5'UTR and mouse DSCR1.4 5'UTR sequence are highly matched.

(A) Used human DSCR1.4 mRNA 5'UTR sequence. (B) Used mouse DSCR1.4 mRNA 5'UTR sequence. The sequence written in red represents formally noted mouse DSCR1.4 5'UTR sequence. The sequence written in black represents 136 genomic DNA sequence in upstream of reported Exon 1 of mDSCR1.4 gene. (C) Alignment data of human DSCR1.4 5'UTR and mouse DSCR1.4 5'UTR. The nucleotides written in blue means consensus sequence.

Supplementary Figure 2. Seo *et al.*

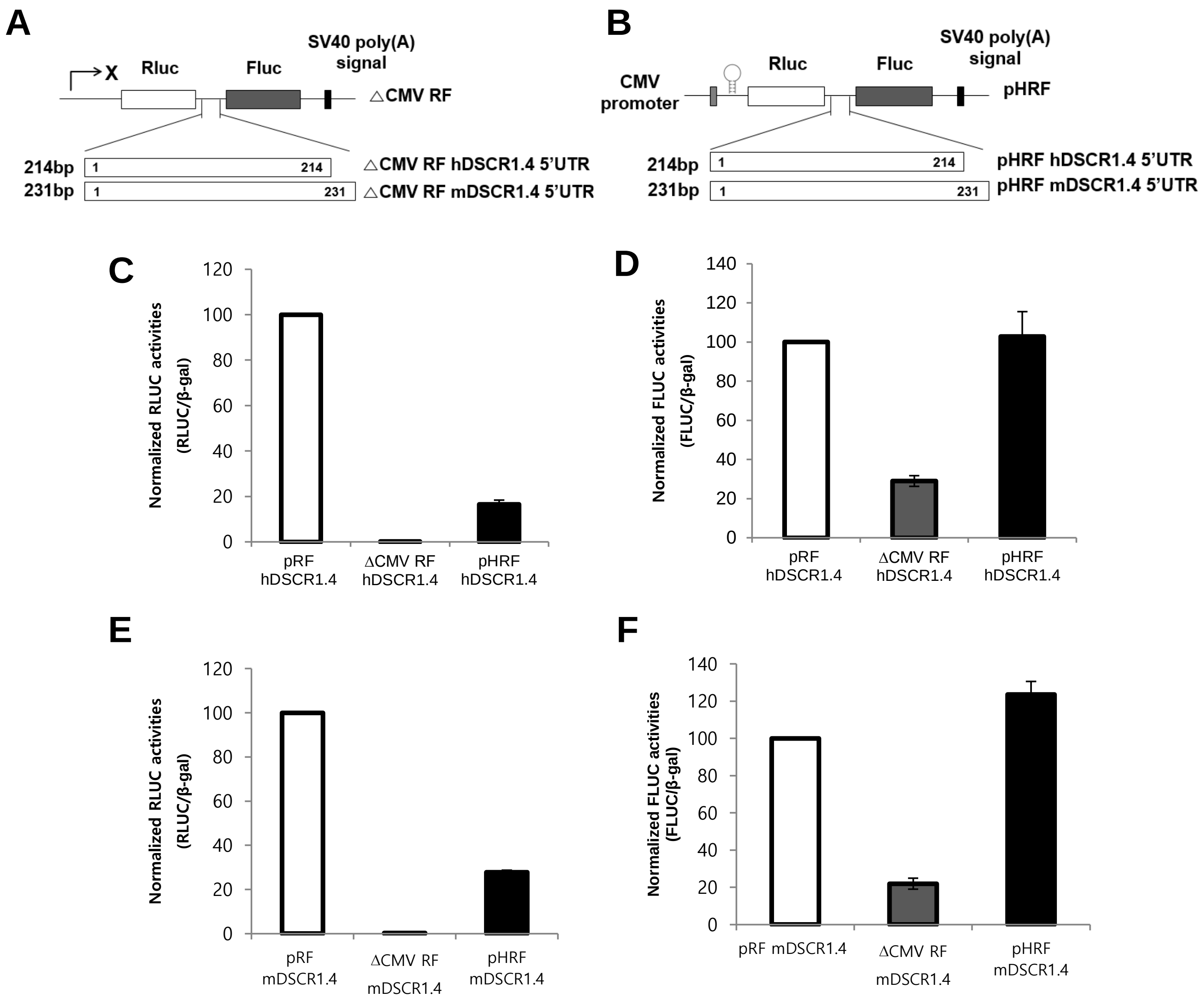

Supplementary Figure 2. DSCR1.4 5'UTR has a cap-independent translation activity.

(A) Schematic representation of the bicistronic reporter vector without CMV promoter (ΔCMV RF vector). (B) Schematic representation of the bicistronic reporter vector with a hairpin structure upstream RLUC cistron (pHRF vector). (C, D) SHSY5Y cells were transfected with pRF, ΔCMV RF, pHRF plasmids with hDSCR1.4 5'UTR. (E, F) N2A cells were transfected with pRF, ΔCMV RF, pHRF plasmids with mDSCR1.4 5'UTR. (C, E) RLUC and (D, F) FLUC activities were normalized by co-transfected β-galactosidase activity. Normalized luciferase activity of pRF vector was set as 100. Student's t-test (n = 5).

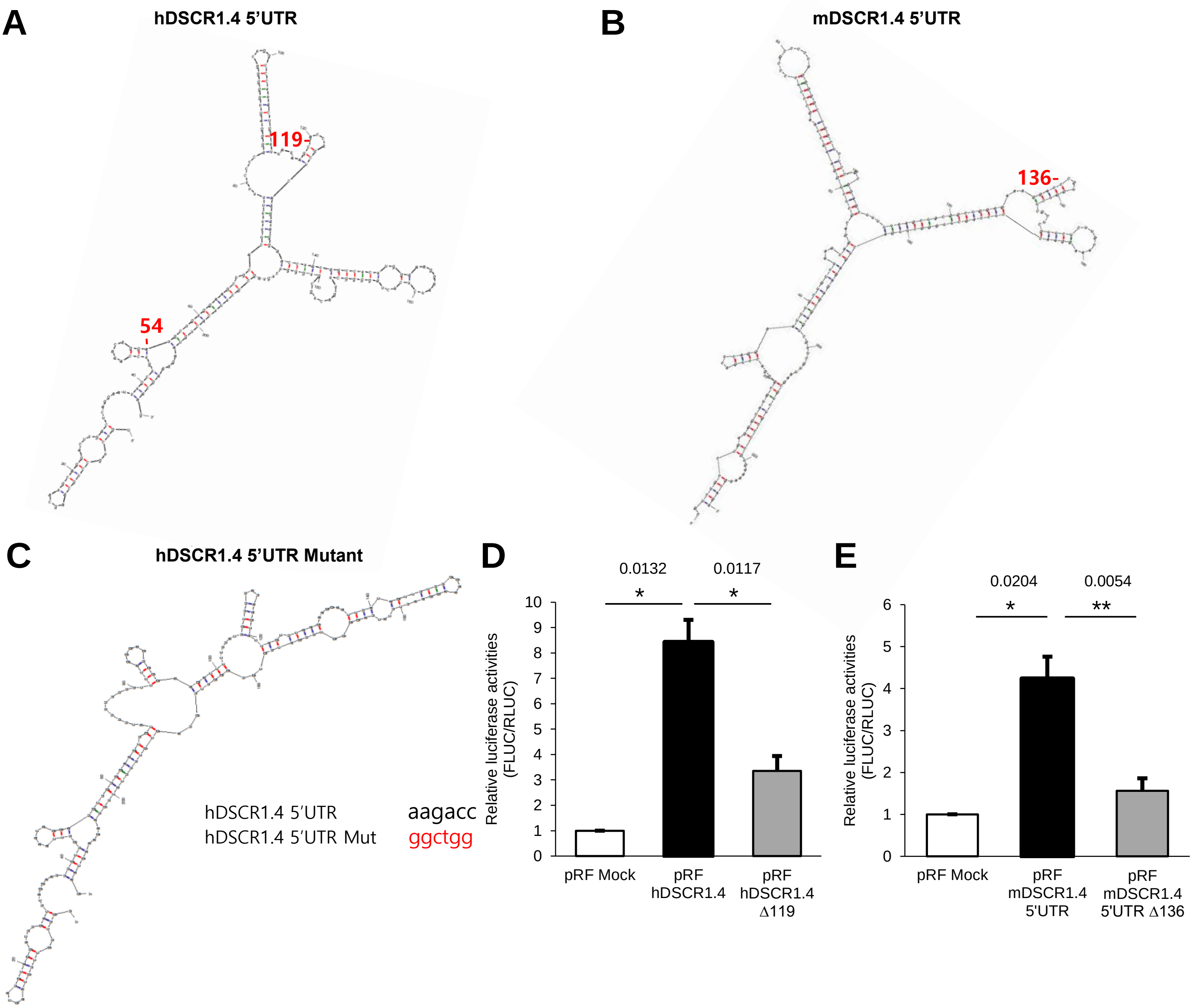

Supplementary Figure 3. Cis-acting region of hDSCR1.4 5'UTR and mDSCR1.4 5'UTR.

(A, B) Predicted RNA secondary structure of 5'UTR of (A) hDSCR1.4 and (B) mDSCR1.4 mRNA. (C) Predicted RNA secondary structure of hDSCR1.4 5'UTR Mutant where 6 nucleotides from 95 to 100 were mutated. (D) The 5' proximal 119 nucleotides sequence of hDSCR1.4 5'UTR is important for cap-independent translation activity of hDSCR1.4 5'UTR. In vitro transcribed RF hDSCR1.4 5'UTR and RF hDSCR1.4 5'UTR Δ119 transcripts were transfected into SHSY5Y cells. The bars represent the mean±SEM (n=3). (E) The 5' proximal 136 nucleotides sequence of mDSCR1.4 5'UTR is essential for cap-independent translation activity of mDSCR1.4 5'UTR. In vitro transcribed RF mDSCR1.4 5'UTR and mDSCR1.4 5'UTR Δ136 transcripts were transfected on N2A cells. The bars represent the mean±SEM (n=4). Data information: In (D, E), *P<0.05, **P<0.01 (Student's t-test).

Supplementary Figure 4. Seo *et al.*

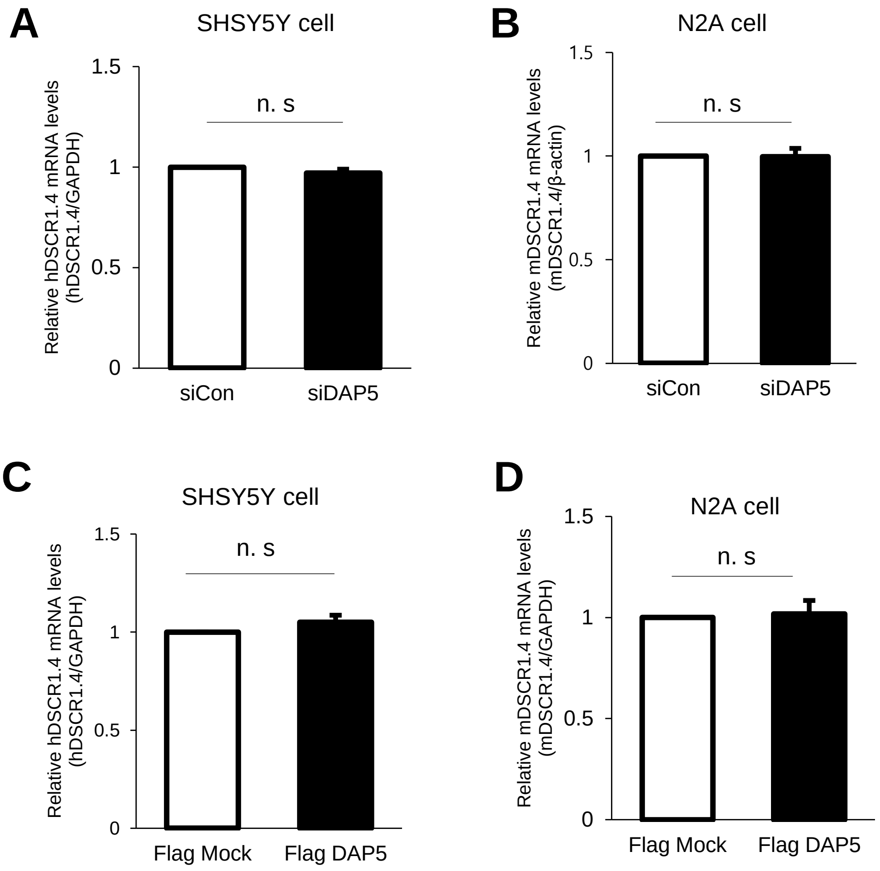

Supplementary Figure 4. Inhibition of DAP5 expression does not change the levels of DSCR1.4 mRNA.

(A, B) DAP5 knock-down does not affect the level of DSCR1.4 mRNA. (A) SHSY5Y and (B) N2A cells were transfected with siCon and siDAP5 and incubated for 24 hours. (C, D) The increased DAP5 expression does not change the level of DSCR1.4 mRNA. (C) SHSY5Y and (D) N2A cells were transfected with Flag Mock and Flag DAP5 plasmids and incubated for 24 hours. Endogenous DSCR1.4 mRNA levels were measured by quantitative real-time PCR (qRT-PCR) and normalized to GAPDH mRNA. The bars represent the mean±SEM (A; n=4, B; n=3, C; n=4, D; n=3). Data information: In (A-D), n.s., non-significant (Student’s t-test).

Supplementary Figure 5. Seo *et al.*

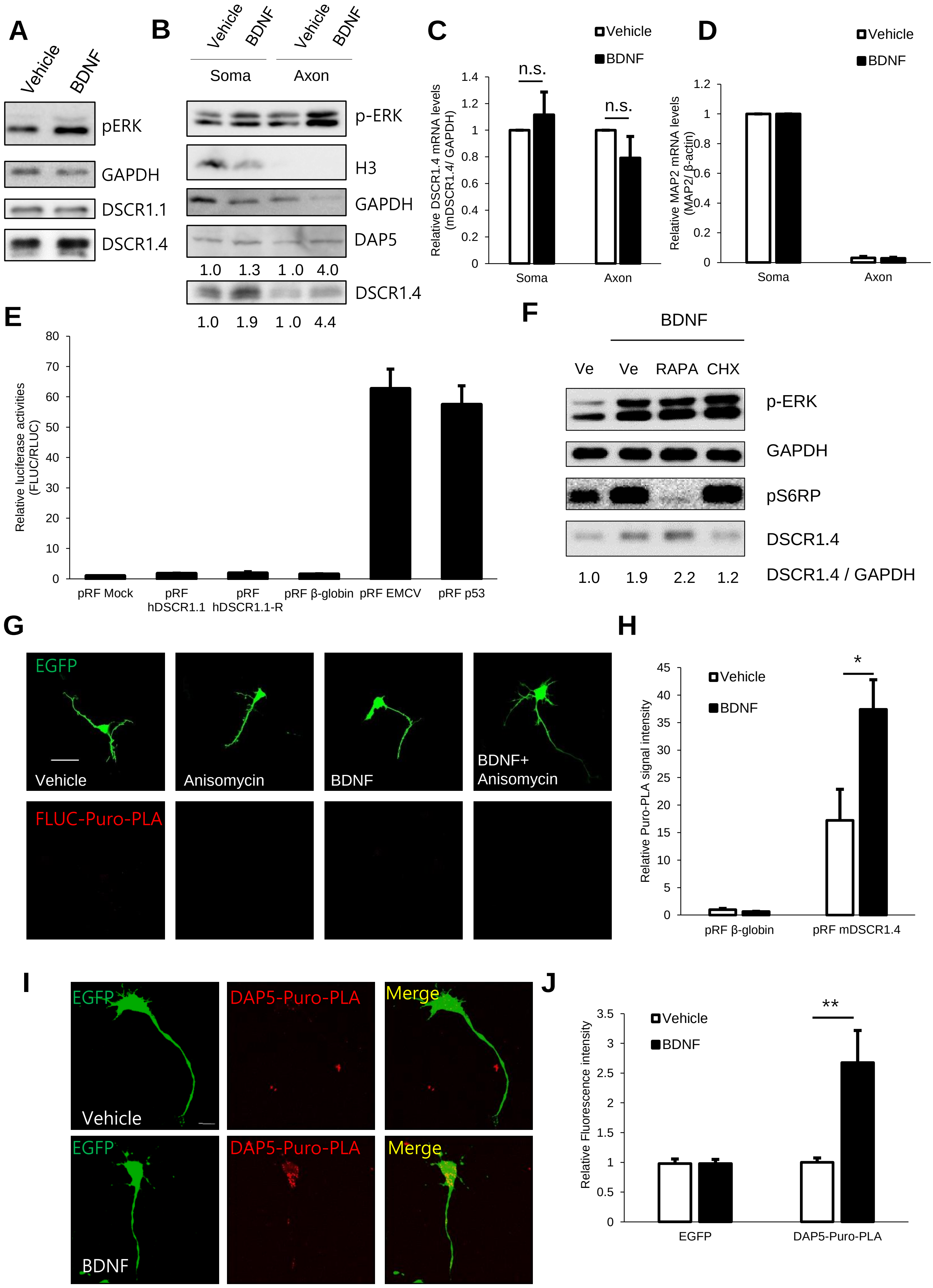

Supplementary Figure 5. BDNF accelerates cap-independent translation of DSCR1.4 mRNA.

(A) BDNF treatment increases protein levels of DSCR1.4 not DSCR1.1. DIV 3 hippocampal neurons were treated with 30μM BDNF for 1 hour. The protein levels were measured by Western blotting. (B) BDNF enhances DSCR1.4 protein expression in both soma and axon of hippocampal neurons. Primary hippocampal neurons were cultured at boyden chamber. After the isolation of axon and soma, the protein levels were measured by Western blotting. Histone H3 was used as soma-enriched marker. The numbers at the bottom indicate the fold relative to the vehicle. The amount of DAP5 and DSCR1.4 was normalized to GAPDH. (C, D) BDNF did not affect the distribution of DSCR1.4 mRNA. (C) After fractionation of soma and axon compartment, endogenous DSCR1.4 mRNA levels were analyzed by qRT-PCR and normalized to GAPDH mRNA. (D) Fractionation of soma and axon compartment was confirmed by soma-enriched marker, MAP2. MAP2 mRNA levels were normalized by β-actin which exists in both soma and axon. The bars represent the mean±SEM (n=3). (E) hDSCR1.1 5'UTR has no cap-independent activity. pRF reporter plasmids were transfected on SHSY5Y cells. The activity of pRF Mock was set to 1. The bars represent the mean±SEM (n=3). (F) Cap-independent translation is essential for DSCR1.4 protein accumulation by BDNF. DIV 3 hippocampal neurons were treated with vehicle (DMSO), 200μM rapamycin (RAPA) or 50mg/ml cycloheximide (CHX) for 3 hours followed by BDNF treatment for 1 hour. The levels of each protein were confirmed by Western blot. The numbers at the bottom indicate the fold change relative to the vehicle. The amount of DSCR1.4 was normalized to GAPDH. (G, H) Cap-independent translation of β-globin mRNA is not induced unlike DSCR1.4 mRNA in soma and axon. EGFP and pRF β-globin 5'UTR vectors were co-transfected into DIV2 mouse hippocampal neurons. At 24 hours later, 100μM anisomycin was treated for 3 hours and then 30μM BDNF was treated for 1 hour, followed by 5μM puromycin treatment for 40 minutes. To detect newly synthesized FLUC proteins, Puro-PLA assay was conducted. (G) Representative image obtained from confocal microscopy. (H) The graph shows relative fluorescence intensity measured by Image J. The bars represent the mean±SEM. Scale bar, 30μm. (I, J) BDNF increases translation of DAP5. EGFP plasmids were transfected into DIV2 mouse hippocampal neurons. At 24 hours later, 30μM BDNF was treated for 1 hour, followed by 5μM puromycin treatment for 40 minutes. To observe newly synthesized endogenous DAP5 proteins, Puro-PLA assay was performed. (I) Representative image. (J) The graph shows relative fluorescence intensity measured by Image J. The bars represent the mean±SEM (Vehicle; n=18, BDNF; n=17). Data information: In (C-E, H, J), **P*<0.05, ***P*<0.01 (Student's t-test).

Supplementary Figure 6. Seo *et al.*

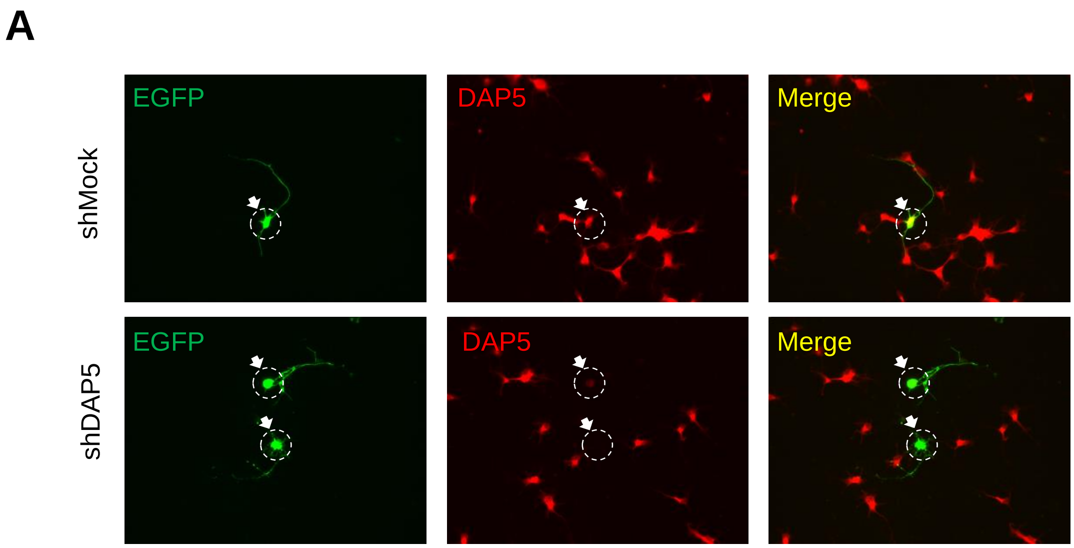

Supplementary Figure 6. DAP5 shRNA inhibits DAP5 expression effectively.

(A) shMock and shDAP5 were transfected on DIV1 hippocampal neuron. After 48 hours, protein levels were confirmed by Immunocytochemistry (ICC). EGFP signal expressing cells are shRNA transfected cells. The arrow means shRNA transfected cell.

Supplementary Figure 7. Seo *et al.*

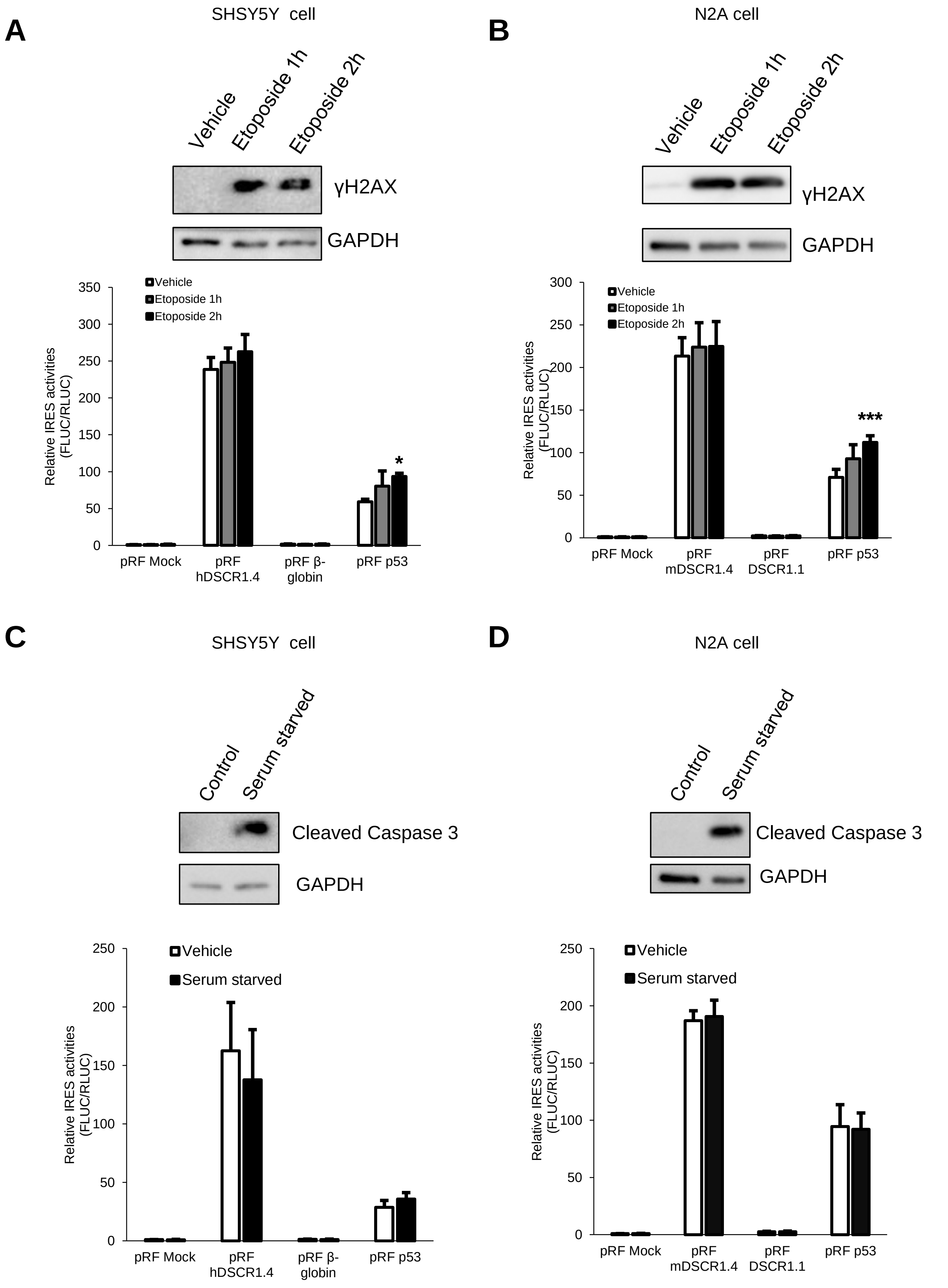

Supplementary Figure 7. DNA-damage and serum deprivation stress do not affect cap-independent translation activity of DSCR1.4 mRNA

(A, B) DNA-damage drug, etoposide treatment does not have an influence on the cap-independent translation activity of DSCR1.4. (A) SHSY5Y and (B) N2A cells were transfected with pRF bicistronic vectors and DMSO or etoposide 100μM was added at 18 h after the transfection for the indicated period of time. Etoposide treatment was confirmed by Western blot. GAPDH and γH2AX were used as a loading control and marker of etoposide-induced DNA-damage, respectively. Luciferase activity of pRF mock transfected and DMSO treated cells was set as 1. The bars represent the mean±SEM (n=3, n=3). (C, D) Serum deprivation stress does not affect the cap-independent translation of DSCR1.4 mRNA. (C) SHSY5Y and (D) N2A cells were transfected with pRF bicistronic vectors and were grown in normal or serum-depleted media for 24 hours. Serum deprivation was confirmed by Western blot. GAPDH and cleaved caspase-3 were used as a loading control and marker of serum deprivation-induced apoptosis, respectively. Luciferase activity of pRF mock transfected cells incubated in normal growth media was set as 1. The bars represent the mean±SEM (n=3, n=3). Data information: In (A-D), n.s., non-significant ,**P*<0.05, ***P*<0.01 (Two-way ANOVA).
